# Long-term dexamethasone treatment diminishes store-operated Ca^2+^ entry in salivary acinar cells

**DOI:** 10.1038/s41368-018-0031-0

**Published:** 2019-01-03

**Authors:** Yuichiro Kusuda, Yusuke Kondo, Yuta Miyagi, Takashi Munemasa, Yusuke Hori, Fumiko Aonuma, Shintaro Tsuka, Taro Mukaibo, Chihiro Masaki, Ryuji Hosokawa

**Affiliations:** Division of Oral Reconstruction and Rehabilitation, Kyushu Dental University, Kitakyushu, Fukuoka 803-8580 Japan

## Abstract

Corticosteroids are used in the treatment of many diseases; however, they also induce various side effects. Dexamethasone is one of the most potent corticosteroids, and it has been reported to induce the side effect of impaired salivary gland function. This study aimed to evaluate the effects of dexamethasone on mouse submandibular gland function to gain insight into the mechanism of dexamethasone-induced salivary hypofunction. The muscarinic agonist carbachol (CCh) induced salivary secretion and was not affected by short-term dexamethasone treatment but was decreased following long-term dexamethasone administration. The expression levels of the membrane proteins Na^+^-K^+^-2Cl^−^ cotransporter, transmembrane member 16A, and aquaporin 5 were comparable between the control and long-term dexamethasone treatment groups. The CCh-induced increase in calcium concentration was significantly lower in the presence of extracellular Ca^2+^ in the long-term dexamethasone treatment group compared to that in the control group. Furthermore, CCh-induced salivation in the absence of extracellular Ca^2+^ and Ca^2+^ ionophore A23187-induced salivation was comparable between the control and long-term dexamethasone treatment groups. Moreover, salivation induced by the Ca^2+^-ATPase inhibitor thapsigargin was diminished in the long-term dexamethasone treatment group. In summary, these results demonstrate that short-term dexamethasone treatment did not impair salivary gland function, whereas long-term dexamethasone treatment diminished store-operated Ca^2+^ entry, resulting in hyposalivation in mouse submandibular glands.

## Corticosteroids: revealing the mechanism of drug-induced dry mouth

Long-term corticosteroid use causes dry mouth by inhibiting calcium movement into salivary gland cells. Corticosteroid use is associated with multiple systemic side effects including salivary inhibition, prompting Yusuke Kondo and a research team from Japan’s Kyushu Dental University to elucidate the reasons why. Submandibular glands taken from mice after six weeks’ treatment with the corticosteroid dexamethasone produced significantly less saliva in ten minutes than a group given just one week’s treatment, or no treatment at all. Further investigation revealed that long-term dexamethasone treatment inhibited the ability of salivary gland cells, known as acinar cells, to transport calcium ions from the exterior to the interior of the cells—an essential mechanism enabling salivation. The study improves our understanding of corticosteroid-induced dry mouth and reveals potential pharmacological targets to enable treatment.

## Introduction

Many medical conditions, including asthma, rheumatoid arthritis, and systemic lupus erythematosus, are treated with corticosteroids, but they also induce many side effects.^1^ Increased risks of infection, osteoporosis, fracture, gastrointestinal bleeding, and many other pathologies have been reported as common and severe side effects.^2,3^ Furthermore, corticosteroid treatment affects systematic metabolism^4^ and decreases the body and organ weights of the liver, thymus, and spleen.^5^

Corticosteroids also affect the oral region. Dexamethasone, a potent glucocorticoid, can cause salivary alterations,^6^ and glucocorticoids increase the frequency of experiencing oral dryness.^7^ In an animal study, dexamethasone-treated rats exhibited significantly reduced salivary secretion.^8,9^ Furthermore, dexamethasone reduced salivary gland mass and increased insulin resistance, which may have a negative impact on salivary gland homeostasis.^9^ However, the mechanism underlying these effects at the cellular level is not well-understood.

Dry mouth is caused by salivary gland dysfunction and results in oral mucosal pain, dysphagia, stomatitis, difficulty wearing dentures, and increased risk of dental caries and periodontal disease.^10,11^ The salivary glands are composed of acinar cells and ductal cells, and many channels and transporters contribute to salivary secretion.^12,13^ Activated muscarinic receptors lead to G protein-regulated activation of phosphatidylinositol 4,5, bisphosphate (PIP2)-specific phospholipase C and PIP2 hydrolysis, which results in the generation of inositol 1,4,5-trisphosphate (IP_3_) and diacylglycerol. IP_3_ activates the IP_3_ receptor, which is expressed on the endoplasmic reticulum (ER) membrane. Activated IP_3_ receptor induces Ca^2+^ release from the ER, which results not only in an increase in intracellular calcium concentrations ([Ca^2+^]_i_) but also in depletion of Ca^2+^ in the ER. Depleted ER Ca^2+^ causes extracellular Ca^2+^ entry, which is referred to as store-operated Ca^2+^ entry (SOCE).^14,15^ An increase in [Ca^2+^]_i_ is essential for salivary fluid secretion, and impaired [Ca^2+^]_i_ increase is associated with salivary hypofunction in patients with Sjögren’s Syndrome.^16^ Increased [Ca^2+^]_i_ induces Cl^−^ movement through the transmembrane protein 16A (TMEM16A) Ca^2+^-activated Cl^−^ channel.^17–19^ The accumulation of Cl^−^ in the lumen induces water movement through the aquaporin 5 (AQP5) water channel and tight junctions. The Na^+^-K^+^-2Cl^−^ cotransporter (NKCC1) and anion exchanger move Cl^−^ into acinar cells, consequently maintaining continuous salivary secretion.^20,21^ NKCC1 also plays a substantial role in determining the amplitude of oscillatory Cl^−^ currents in salivary acinar cells.^22^ Furthermore, the ductal epithelial Na^+^ channel and cystic fibrosis transmembrane conductance regulator aid in Na^+^ and Cl^−^ reabsorption from saliva, respectively.

Previous reports have demonstrated that glucocorticoids affect the expression or function of channels and transporters in some tissues. For example, dexamethasone increased AQP1 expression and water transport in rat peritoneum.^23^ Moreover, NKCC1 expression was reduced by glucocorticoids in alveolar and bronchial cells.^24^ Furthermore, dexamethasone treatment decreased [Ca^2+^]_i_ in dendritic cells,^25^ pancreatic β-cells,^26^ and bronchial epithelial cells.^27^ Another study reported that dexamethasone decreased [Ca^2+^]_i_ and consequently inhibited Cl^−^ secretion in human bronchial epithelial cells.^28^ Therefore, we hypothesized that dexamethasone may directly affect salivary acinar cell function and that the expression of channels and transporters in submandibular acinar cells (e.g., TMEM16A, AQP5, and NKCC1) or intracellular Ca^2+^ signalling may be impaired by dexamethasone treatment, which is true for other cells and tissues,^25,29,30^ resulting in dry mouth. However, the effects of dexamethasone on channels, transporters, and intracellular Ca^2+^ signalling in salivary glands are currently unknown.

## Results

### Gland weights and blood glucose levels

**Fig. 1:**
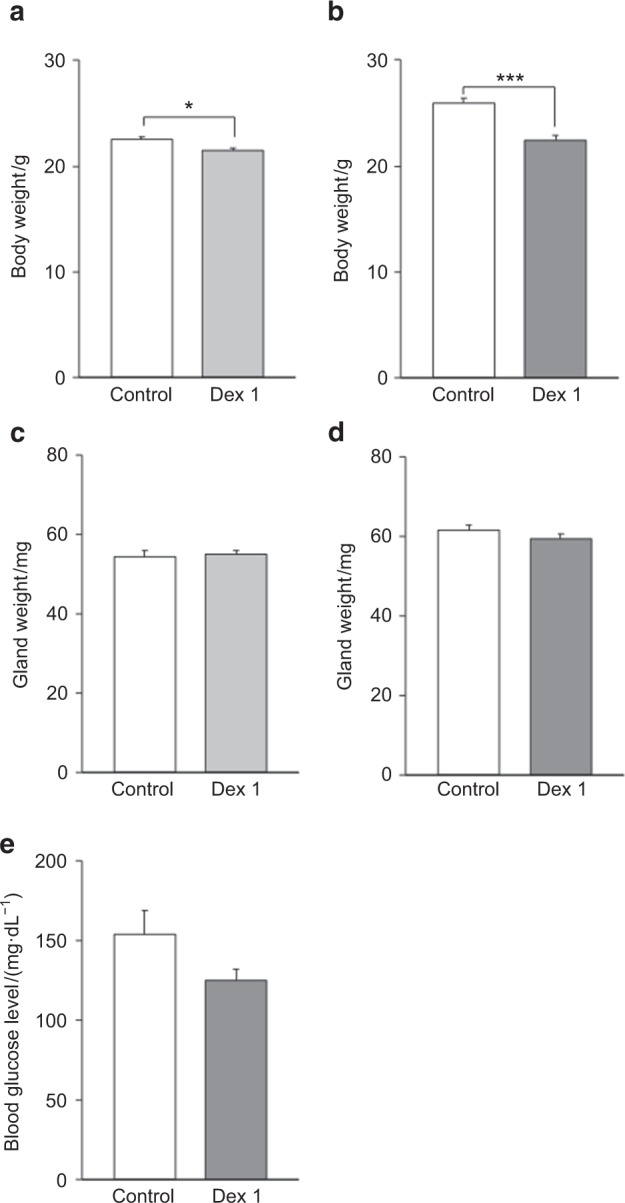
Body weights, gland weights, and blood glucose levels. The effects of dexamethasone treatment on body weight (**a**, **b**), submandibular gland (SMG) weight (**c**, **d**), and blood glucose levels (**e**). **a** Body weights were comparable between the control and Dex1 groups. **b** Body weights were significantly lower in the Dex6 group than in the control group (*n* = 14 per group). **c** The SMG weights were comparable between the control and Dex1 groups (*n* = 12 and 8, respectively). **d** The SMG weights were comparable between the control and Dex6 groups (*n* = 28 per group). **e** There was no significant difference in blood glucose levels between the control and Dex6 groups (*n* = 4 per group). Data represent the mean ± SE; ***P < 0.001

As shown in Figs. 1a, b, body weight was significantly lower in the Dex1 and Dex6 groups than in the control group (Figs. 1a, b). Moreover, gland weights were comparable among the Dex1, Dex6, and control groups (Figs. 1c, d). Dexamethasone treatment did not increase blood glucose levels (Fig. 1e).

### Salivary secretion from ex vivo SMGs and increased [Ca^2+^]_i_ in SMG acinar cells

**Fig. 2:**
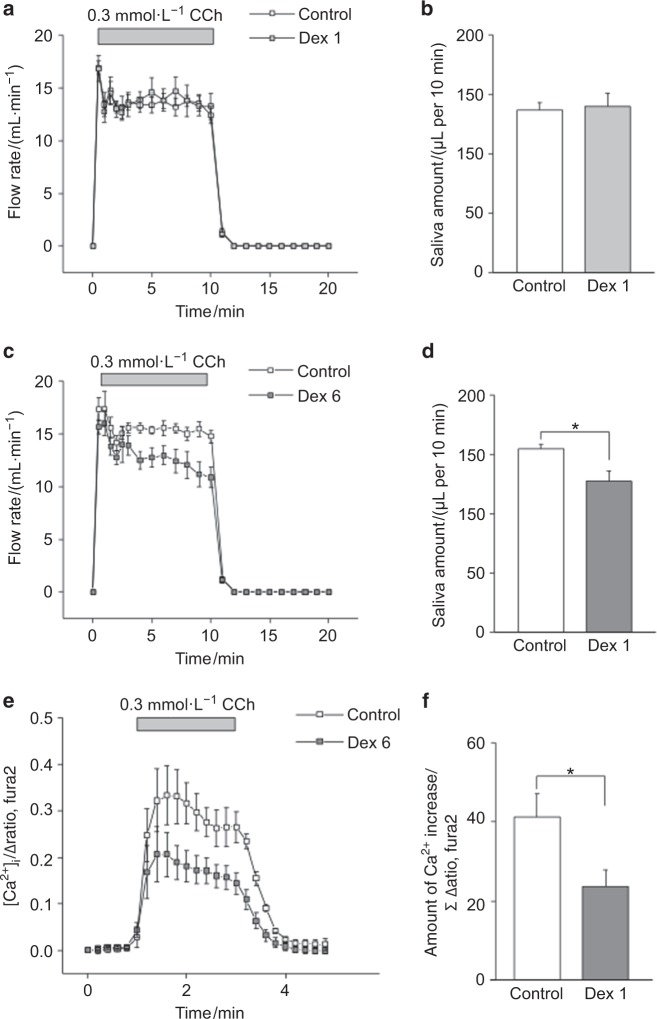
Salivary secretion from ex vivo SMGs and increased calcium concentration ([Ca^2+^]_i_) in SMG acinar cells. The muscarinic agonist carbachol (CCh)-induced salivary secretion from ex vivo SMGs and the increased [Ca^2+^]_i_ in Fura 2-AM-loaded acinar cells were measured. **a**,** b** The CCh-induced salivary secretion rate and quantity of saliva produced in 10 min were comparable between the control and Dex1 groups (*n* = 7 per group). **c**,** d** The CCh-induced salivary secretion rate was lower in the Dex6 group (**c**), and the quantity of saliva produced in 10 min was significantly lower in the Dex6 group than in the control group (**d**) (*n* *=* 7 and 8, respectively). **e**,** f** The CCh-induced [Ca^2+^]_i_ increase in SMG acinar cells was significantly lower in the Dex6 group than in the control group (*n* = 3 and 6, respectively). *P*-values were obtained using one-way ANOVA followed by Bonferroni’s post hoc test (**b**,** d**) and Student’s *t*-test (**f**). Data represent the mean ± SE; *P < 0.05

CCh, a muscarinic agonist, and A23187, a Ca^2+^ ionophore, induced salivation in ex vivo SMGs. The CCh-induced salivary flow rate is shown in Figs. 2a, c, and the amounts of saliva are shown in Figs. 2b, d. The amount of saliva produced in 10 min was significantly lower in the Dex6 group than in the control group (Fig. 2d) but was comparable between the Dex1 and control groups (Fig. 2b). We measured the CCh-induced [Ca^2+^]_i_ increase in Fura 2-AM-loaded SMG acinar cells (Fig. 2e). [Ca^2+^]_i_ was significantly decreased in the Dex6 group, suggesting that the weaker increase in [Ca^2+^]_i_ decreased salivary secretion in the Dex6 group (Fig. 2f).

### Calcium ionophore-induced [Ca^2+^]_i_ increase and salivation from ex vivo SMGs

**Fig. 3:**
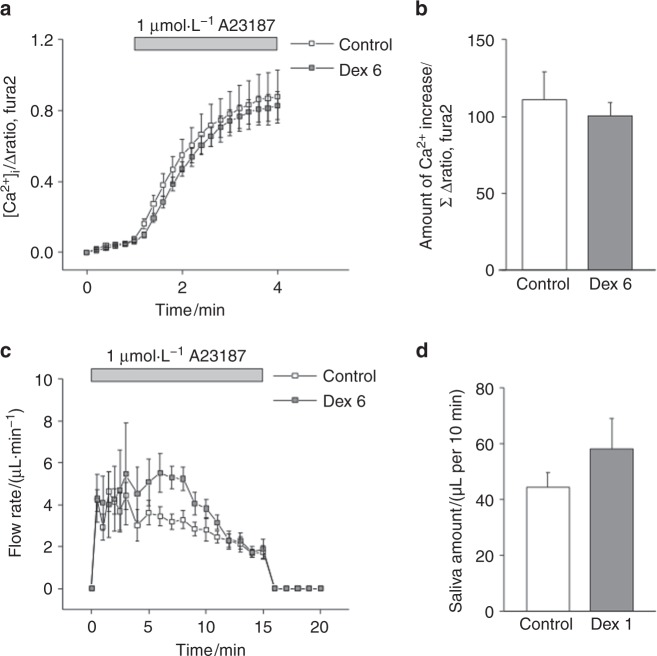
The Ca^2+^ ionophore-induced [Ca^2+^]_i_ increase and salivary secretion from ex vivo SMGs. **a**,** b** The A23187-induced [Ca^2+^]_i_ increase in SMG acinar cells was comparable between the control and Dex6 groups (*n* = 9 and 8, respectively). **c**,** d** The Ca^2+^ ionophore A23187-induced salivary flow rate is shown. There was no significant difference in the amount of A23187-induced saliva between the control and Dex6 groups (*n* = 5 and 4, respectively). Data represent the mean ± SE

Calcium ionophores are assumed to directly facilitate the transport of Ca^2+^ across the plasma membrane, which results in an increase in [Ca^2+^]_i_. First, we measured the [Ca^2+^]_i_ increase in response to A23187, a calcium ionophore, which resulted in comparable calcium ionophore-induced [Ca^2+^]_i_ levels between the control and Dex6 groups (Figs. 3a, b). To evaluate the salivary secretion in response to a certain level of [Ca^2+^]_i_ increase, the salivary flow rate induced by A23187 was measured. Figure 3c shows the A23187-induced salivary flow rates, and the amount of saliva produced is shown in Fig. 3d. A23187 induced salivation in the SMGs in both the control and Dex6 groups (Fig. 3c), and we did not observe any significant difference in the amount of saliva produced between the Dex6 and control groups (Fig. 3d).

### Histological analysis and expression of membrane proteins in the SMG

**Fig. 4:**
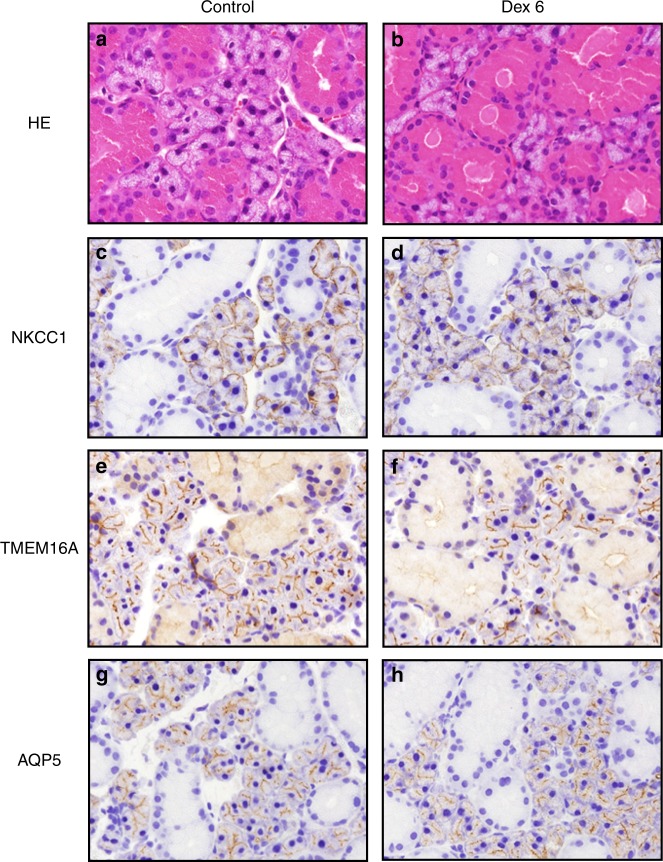
Histological analysis and expression of membrane proteins in SMGs. **a**,** b** The morphological evaluation of control and Dex6 SMGs was conducted using haematoxylin and eosin (HE)-stained images. **c**-** h** The expression of three critical membrane proteins for salivary secretion, TMEM16A (**c** and **d**), AQP5 (**e** and **f**), and NKCC1 (**g** and **h**), was evaluated using immunohistochemistry. TMEM16 and AQP5 were expressed in the acinar apical membrane, and NKCC1 was expressed in the acinar basolateral membrane in both control and Dex6 SMGs

Morphological evaluation was conducted using HE-stained images. As shown in Fig. 4, we did not observe inflammatory cell infiltration or a fibrotic response in the Dex6 group (Fig. 4), suggesting that inflammatory cell infiltration did not occur in dexamethasone-treated SMGs. Immunohistochemistry was used to detect three major membrane proteins in SMG acinar cells: TMEM16A, AQP5, and NKCC1. TMEM16 and AQP were expressed at the acinar apical membrane, and NKCC1 was expressed at the acinar basolateral membrane in both the control and Dex6 groups (Fig. 4).

### NKCC1 activity in SMG acinar cells

**Fig. 5:**
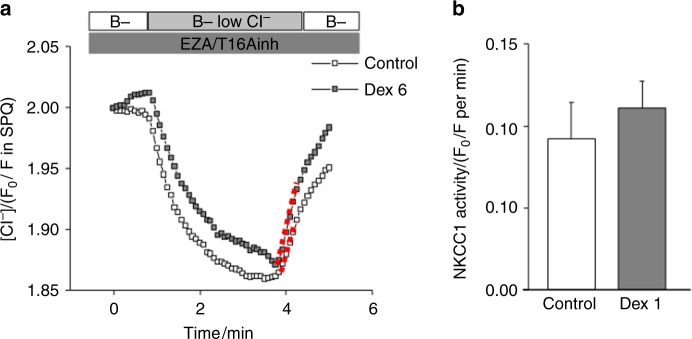
NKCC1 activity in acinar cells. Cl^−^ uptake experiments were performed in SPQ-loaded acinar cells. HCO_3_^-^-free solutions with EZA and T16Ainh-A01 were used. **a** Intracellular Cl^−^ was depleted by a low Cl^−^ solution, and then Cl^−^ uptake was initiated by the addition of high Cl^−^ solution in control and Dex6 acinar cells (*n* = 5 and 6, respectively). **b** A summary of the Cl^−^ uptake rates calculated from experiments similar to those shown in **a**; Cl^−^ uptake rates were comparable between the control and Dex6 groups (*n* = 5 and 6, respectively)

Next, the function of NKCC1 was evaluated. As described in the Materials and Methods section, the Cl^−^ influx rate represents NKCC1 activity (Fig. 5a). The Cl^−^ influx rate was comparable between the control and Dex6 groups, demonstrating that NKCC1 activity was not affected by long-term dexamethasone treatment (Fig. 5b).

### Extracellular Ca^2+^-independent or Ca^2+^-ATPase inhibitor-induced [Ca^2+^]_i_ increase and salivary secretion from ex vivo SMGs

**Fig. 6:**
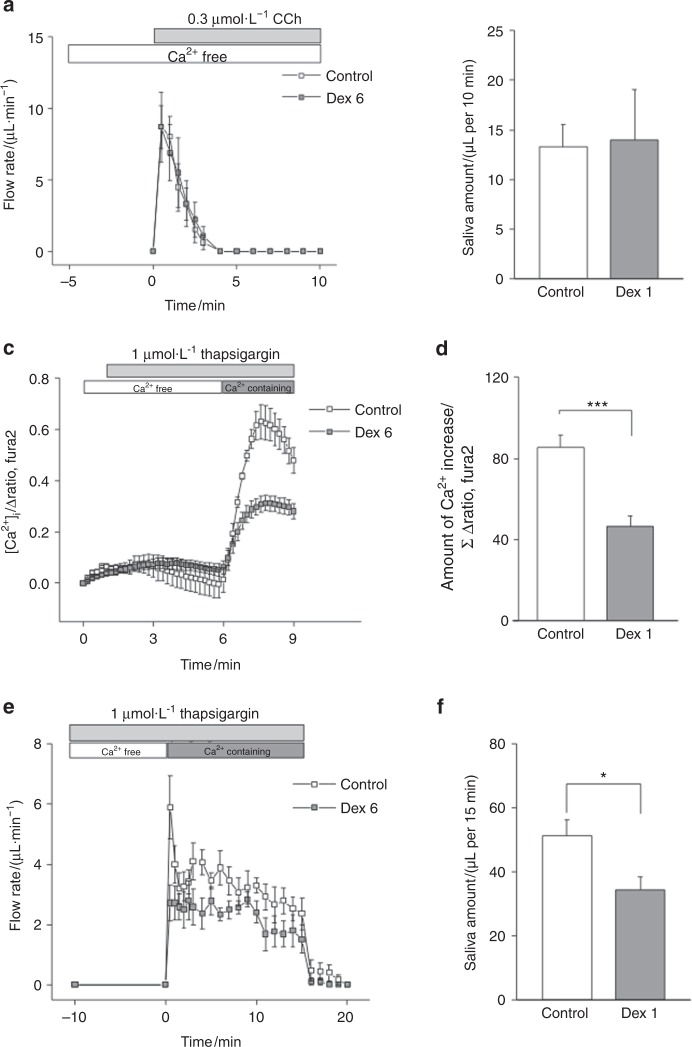
Extracellular Ca^2+^-independent salivation, and Ca^2+^-ATPase inhibitor-induced [Ca^2+^]_i_ increase and salivation in ex vivo SMGs. CCh- and thapsigargin-induced salivary secretion was measured in ex vivo mouse SMGs. **a**,** b** CCh-induced salivary secretion under extracellular Ca^2+^-free conditions is shown. The amount of saliva was comparable between the control and Dex6 groups (*n* = 4 and 5, respectively). **c**,** d** The thapsigargin-induced [Ca^2+^]_i_ increase in SMG acinar cells in the presence of extracellular Ca^2+^ is shown. The amount of [Ca^2+^]_i_ increase was significantly lower in the Dex6 group than in the control group (*n* = 6 and 8, respectively). **e**,** f** The amount of saliva induced by thapsigargin, a Ca^2+^-ATPase inhibitor, was measured. The amount of saliva induced by thapsigargin in 15 min was significantly lower in the Dex6 group than in the control group (*n* = 8 and 6, respectively). Data represent the mean ± SE; ***P < 0.001

The above results suggest that dexamethasone treatment affected the mechanism of [Ca^2+^]_i_ increase in SMG acinar cells, consequently diminishing salivary secretion. To investigate the mechanism in greater detail, we removed extracellular Ca^2+^ in ex vivo SMGs. Without extracellular Ca^2+^, CCh-induced salivation commenced as usual and decreased after 2 min (Fig. 6a), and the amount of secreted saliva was comparable between the Dex6 and control groups (Fig. 6b). Then, the [Ca^2+^]_i_ increase and saliva production induced by the Ca^2+^-ATPase inhibitor thapsigargin was measured to evaluate SOCE. The thapsigargin-induced [Ca^2+^]_i_ increase is shown in Fig. 3c. Thapsigargin causes an increase in [Ca^2+^]_i_ in the presence of extracellular Ca^2+^; the amount of [Ca^2+^]_i_ increase is shown in Fig. 3d. The amount of [Ca^2+^]_i_ increase was significantly lower in the Dex6 group than in the control group (Fig. 3d). No saliva was secreted following thapsigargin treatment in the absence of extracellular Ca^2+^, but saliva secretion occurred when Ca^2+^ was added to the extracellular solution (Fig. 6e). Thapsigargin-induced saliva production at 15 min was significantly lower in the Dex6 group than in the control group (Fig. 6f), suggesting that extracellular Ca^2+^-dependent salivation was affected by long-term dexamethasone treatment.

## Discussion

We found that long-term dexamethasone treatment significantly decreased salivation from ex vivo mouse SMGs, as shown in Fig. 2d, whereas short-term dexamethasone treatment did not have an effect on salivation, and we endeavoured to reveal the details of this mechanism. First, we examined dexamethasone treatment-induced morphological changes in SMGs. However, SMG weights were comparable between the control and Dex6 groups, and we did not find any apparent differences in the HE-stained histological images between the groups, suggesting that dexamethasone-induced salivary hypofunction was not due to any morphological effects. Glucocorticoids have been reported to significantly affect systematic metabolism^4^ and increase body weight in humans, whereas earlier reports demonstrated that dexamethasone treatment decreased body weight in mice,^5^ which was consistent with our results.

We also examined the expression of TMEM16A, AQP5, and NKCC1 using immunohistochemistry. The expression of these membrane proteins was comparable between the control and dexamethasone treatment groups (Fig. 4), suggesting that dexamethasone did not affect membrane protein expression in this study. We tried to quantify the protein levels of TMEM16A, AQP5, and NKCC1 by western blot; however, it was difficult to separate SMGs into acinar cells and duct cells for the analysis. In particular, we observed TMEM16A expression in not only the acinar cells but also the duct cells. The activity of these membrane proteins was then evaluated. Regarding NKCC1, we measured its activity using ion imaging, which revealed that long-term dexamethasone treatment did not impair NKCC1 activity (Figs. 5a, b). We did not measure TMEM16A and AQP5 activity directly; therefore, the calcium ionophore results could be substituted. The calcium ionophore-induced [Ca^2+^]_i_ increase was comparable between the control and Dex6 groups (Figs. 3a, b), and salivary secretion was also comparable between the control and Dex6 groups (Figs. 3c, d), suggesting that TMEM16A and AQP5 activities were also comparable between these two groups.

Diabetes is a known side effect of dexamethasone,^31–33^ and increased blood glucose levels cause hyposalivation.^34,35^ Nevertheless, as shown in Figs. 1e, f, we did not observe blood glucose elevation in the present study. The detailed mechanism is unknown, but we speculate that the difference may be associated with the method of treatment administration. Many previous studies applied intramuscular or intraperitoneal injections, whereas we added dexamethasone to drinking water to minimize stress on the mice. Consequently, we found that salivary hypofunction following dexamethasone treatment was not completely dependent on dexamethasone-induced diabetes.

The most important finding in this study was that long-term dexamethasone treatment decreased SOCE into salivary acinar cells, which resulted in hyposalivation. Previous studies have shown that dexamethasone affects SOCE; however, the effects differed depending on the cell type. SOCE was enhanced by dexamethasone in skeletal muscle cells,^36^ whereas SOCE was impaired in dendritic cells^25^ and pulmonary arterial endothelial cells.^27^ In pulmonary arterial endothelial cells, the expression of FKBP51, a large molecular weight FK506-binding immunophilin, was upregulated by dexamethasone treatment, resulting in enhanced SOCE.^27^ FKBP51 and FKBP52 reportedly regulate various aspects of steroid hormone receptor signalling, including receptor maturation, nuclear translocation, and hormone binding, and FKBP51 is considered a negative regulator of receptor function.^37^ The FKBP51 gene is expressed in human salivary glands.^38^ These reports, together with our findings, suggest that FKBP51 is involved in the mechanism described herein. In conclusion, the present study demonstrated that long-term dexamethasone treatment diminished SOCE into SMG acinar cells, which decreased salivary secretion from SMGs. These findings will be of benefit for the development of new treatment methods for corticosteroid-induced dry mouth.

## Materials and methods

### Materials and animals

Male C57BL/6J adult mice (8 weeks of age) were purchased from CLEA Japan (Tokyo, Japan). Mice were maintained under a 12-h light/dark cycle and fed ad libitum. All experiments were approved by the Animal Committee of Kyushu Dental University (No. 16–011). Collagenase L was purchased from Nitta Gelatin (Osaka, Japan); Fura 2-AM was purchased from Dojindo Molecular Technologies, Inc. (Kumamoto, Japan); 6-methoxy-N-(3-sulfopropyl) quinolinium (SPQ) was purchased from Life Technologies Corporation (Eugene, OR, USA); T16Ainh-A01 was obtained from Tocris Bioscience (Bristol, UK); the NKCC1 antibody (sc-21545) was obtained from Santa Cruz Biotechnology (Dallas, TX, USA); the TMEM16A antibody (ab53213) was purchased from Abcam (Cambridge, UK); the AQP5 antibody (sc-9890) was purchased from EMD Millipore (Billerica, MA, USA); and the secondary antibody (Histofine Simple Stain Mouse MAX peroxidase) was purchased from Nichirei (Tokyo, Japan). All other reagents were purchased from Sigma-Aldrich Japan (Tokyo, Japan).

### Study design

Ex vivo perfusion of the submandibular gland (SMG), ion imaging in dispersed SMG acinar cells and histological analysis studies were performed using a comparative study design. We evaluated the expression of three membrane proteins, TMEM16A, AQP5, and NKCC1, using immunohistochemistry. In the experimental group, dexamethasone was administered via drinking water for 1 week (short-term treatment: Dex1) or 6 weeks (long-term treatment: Dex6), while the control groups received distilled water. The Dex1 and Dex6 groups were composed of different animals. The control groups that were established for the Dex1 and Dex6 groups were also composed of different animals. The Dex1 and Dex6 treatment groups were compared with their respective control groups. In a preliminary experiment, the amount of water intake per day was measured to be approximately 4.7 mL per day. We converted the maximum human dose for articular rheumatism, 8 mg perday, to the corresponding dose in mice based on body surface area. We thus determined the dexamethasone concentration to be 8 mg·L^−1^.

### Perfused solutions

Solutions were as follows (in mmol·L^−1^): HCO_3_^−^ and Ca^2+^-containing solution (B+): 4.3 KCl, 120 NaCl, 25 NaHCO_3_, 5 glucose, 10 HEPES, 1 CaCl_2_, and 1 MgCl_2_, equilibrated with 95% O_2_ and 5% CO_2_; HCO_3_^-^-containing and Ca^2+^-free solution: 4.3 KCl, 122 NaCl, 25 NaHCO_3_, 5 glucose, 10 HEPES, and 1 MgCl_2_, equilibrated with 95% O_2_ and 5% CO_2_; HCO_3_^−^-free and high Cl^−^-containing solution (B-): 4.3 KCl, 120 NaCl, 25 Na gluconate, 5 glucose, 10 HEPES, and 1 MgCl_2_, equilibrated with 100% O_2_; and HCO_3_^−^-free and low Cl^−^ solution: 4.3 K gluconate, 122 Na gluconate, 25 NaHCO_3_, 5 glucose, 10 HEPES, and 1 MgCl_2_, equilibrated with 100% O_2_.

### Blood glucose measurements

Venous blood was drawn from the tail vein. Blood glucose levels were measured using the glucose dehydrogenase flavin adenine dinucleotide method (FreeStyle Freedom Lite glucose monitor; Abbott Japan, Chiba, Japan).

### Ex vivo perfusion of mouse SMGs

Perfusion of the SMG was performed as reported previously.^39^ In brief, mice were anaesthetized with chloral hydrate (400 mg·kg^−1^, i.p.), and the maxillary artery, superficial temporal artery, lingual artery, thyrocervical artery, and internal carotid artery were ligated. Next, the common carotid artery was ligated and cut at the proximal portion of the internal and external carotid branch, and the SMG was removed along with the common carotid artery and duct. The carotid artery was immediately cannulated and perfused with physiological saline solution (B+as described above) at a flow rate of 1 mL·min^−1^ at 37 °C. The perfused SMG was stimulated with a muscarinic agonist (carbachol [CCh]; 0.3 µmol·L^−1^), calcium ionophore (A23187; 1 µmol·L^−1^), or Ca^2+^-ATPase inhibitor (thapsigargin; 1 µmol·L^−1^), and the secreted saliva was collected into a glass capillary tube.

### Histological analysis and immunohistochemistry

For histological analysis, the excised SMGs were fixed in 4% paraformaldehyde phosphate buffer solution for 48 h before being embedded in paraffin. The sections (5-µm thick) were deparaffinized and rehydrated and stained with haematoxylin and eosin (HE). Immunohistochemistry for NKCC1, TMEM16A, and AQP5 was performed as previously described.^34,40^ Briefly, 5-µm thick sections were deparaffinized and rehydrated, and the antigens were unmasked with pH 6.0 citrate buffer solution at 95 °C for 20 min. Endogenous peroxidase activity was inhibited using 0.1% H_2_O_2_. The sections were then incubated for 60 min with an anti-peptide antibody against TMEM16A (1:1), AQP5 (1:200), or NKCC1 (1:1 000), followed by a secondary antibody at room temperature for 30 min to detect bound immunoglobulins, which were visualized using 3,3‵-diaminobenzidine solution at room temperature for 5 min. The sections were then stained with haematoxylin for 1.5 min. Finally, the sections were dehydrated in ethanol and mounted.

### Measurement of [Ca^2+^]_i_

SMGs were removed from the mice after they were anaesthetized with chloral hydrate (400 mg·kg^−1^, i.p.). Acinar cells were isolated by collagenase treatment with 520 U·mL^−1^ collagenase L for 15 min at 37 °C. The isolated cells were incubated with 2 µmol·L^−1^ Fura 2-AM for 10 min at 37 °C. Fluorescence was detected under a microscope equipped with a fluorescence analysis system (Aquacosmos; Hamamatsu Photonics, Hamamatsu, Japan) at an excitation wavelength of 340 or 380 nm and an emission capture of 510 nm. The [Ca^2+^]_i_ was determined as the fluorescence ratio at 340/380 nm. The degree of [Ca^2+^]_i_ increase during the stimulation periods was calculated from the integral value of [Ca^2+^]_i_ as the area under the curve.

### Evaluation of NKCC1 activity in SPQ-loaded acinar cells

The isolated cells were incubated with 5 mmol·L^−1^ SPQ, a Cl^−^ indicator, for 25 min at 37 °C. Fluorescence was detected under a microscope equipped with a fluorescence analysis system (Aquacosmos; Hamamatsu Photonics, Hamamatsu, Japan) at an excitation wavelength of 340 nm and an emission capture of 510 nm. NKCC1 activity was examined in SPQ-loaded SMG acinar cells as previously described.^40^

In brief, extracellular Cl^−^ removal induced Cl^−^ efflux, and extracellular Cl^−^ restoration induced Cl^−^ influx into the acinar cells. Note that the Cl^−^ influx rate represents the NKCC1 activity in the presence of T16Ainh-A01, a TMEM16A inhibitor, and 6-ethoxy-2-benzothiazolesulfonamide (EZA), a carbonic anhydrase inhibitor, and in the absence of HCO_3_^-^ because NKCC1 is the exclusive pathway for Cl^−^ influx under this condition.

### Statistical analysis

Differences between the control and experimental groups were evaluated using the unpaired Student’s *t*-test. Multiple-sample comparisons were performed using one-way ANOVA followed by Bonferroni’s post hoc test. *P* < 0.05 was considered statistically significant. The results are expressed as the mean ± standard error of the mean(SE). All experiments were performed using at least three different mice for each condition, with *n* referring to the number of experiments performed.

## References

[1] Trence DL (2003). Management of patients on chronic glucocorticoid therapy: an endocrine perspective. Prim. Care.

[2] Dixon WG, Suissa S, Hudson M (2011). The association between systemic glucocorticoid therapy and the risk of infection in patients with rheumatoid arthritis: systematic review and meta- analyses. Arthritis Res. Ther..

[3] Costello R, Patel R, Humphreys J, McBeth J, Dixon WG (2017). Patient perceptions of glucocorticoid side effects: a cross-sectional survey of users in an online health community. BMJ Open.

[4] Bordag N (2015). Glucocorticoid (dexamethasone)-induced metabolome changes in healthy males suggest prediction of response and side effects. Sci. Rep..

[5] Rooman R, Koster G, Bloemen R, Gresnigt R, van Buul-Offers SC (1999). The effect of dexamethasone on body and organ growth of normal and IGF-II-transgenic mice. J. Endocrinol..

[6] Sreebny LM, Schwartz SS (1997). A reference guide to drugs and dry mouth. Gerodontology.

[7] Smidt D, Torpet LA, Nauntofte B, Heegaard KM, Pedersen AM (2011). Associations between oral and ocular dryness, labial and whole salivary flow rates, systemic diseases and medications in a sample of older people. Commun. Dent. Oral. Epidemiol..

[8] Johnson DA, Etzel KR, Alvares OF, Cortez JE (1987). Regulation of parotid salivary proteins by glucocorticoids. J. Dent. Res..

[9] Bighetti BB (2014). Long-term dexamethasone treatment alters the histomorphology of acinar cells in rat parotid and submandibular glands. Int. J. Exp. Pathol..

[10] Edgar WM (1992). Saliva: its secretion, composition and functions. Br. Dent. J..

[11] Humphrey SP, Williamson RT (2001). A review of saliva: normal composition, flow, and function. J. Prosthet. Dent..

[12] Nauntofte B (1992). Regulation of electrolyte in salivary acinar cells. Am. J. Physiol. Gastrointest. Liver Physiol..

[13] Melvin JE, Yule D, Shuttleworth T, Begenisich T (2005). Regulation of fluid and electrolyte secretion in salivary gland acinar cells. Annu. Rev. Physiol..

[14] Putney JW (1990). Receptor-regulated calcium entry. Pharmacol. Ther..

[15] Ambudkar IS (2016). Calcium signalling in salivary gland physiology and dysfunction. J. Physiol..

[16] Enger TB, Aure MH, Jensen JL, Galtung HK (2014). Calcium signaling and cell volume regulation are altered in Sjögren’s syndrome. Acta Odontol. Scand..

[17] Romanenko VG (2010). Tmem16A encodes the Ca2+-activated Cl- channel in mouse submandibular salivary gland acinar cells. J. Biol. Chem..

[18] Catalán MA (2015). A fluid secretion pathway unmasked by acinar-specific Tmem16A gene ablation in the adult mouse salivary gland. Proc. Natl. Acad. Sci. USA.

[19] Ambudkar IS (2014). Ca²^+^ signaling and regulation of fluid secretion in salivary gland acinar cells. Cell Calcium.

[20] Evans RL (2000). Severe impairment of salivation in Na+/K+/2Cl- cotransporter (NKCC1)-deficient mice. J. Biol. Chem..

[21] Krane CM (2001). Salivary acinar cells from aquaporin 5-deficient mice have decreased membrane water permeability and altered cell volume regulation. J. Biol. Chem..

[22] Sugita M, Hirono C, Shiba Y (2004). Gramicidin-perforated patch recording revealed the oscillatory nature of secretory Cl- movements in salivary acinar cells. J. Gen. Physiol..

[23] Stoenoiu MS (2003). Corticosteroids induce expression of aquaporin-1 and increase transcellular water transport in rat peritoneum. J. Am. Soc. Nephrol..

[24] Laube M, Bossmann M, Thome UH (2015). Glucocorticoids distinctively modulate the CFTR channel with possible implications in lung development and transition into extrauterine life. PLoS One.

[25] Heise N (2011). Effect of dexamethasone on Na+/Ca2+ exchanger in dendritic cells. Am. J. Physiol. Cell. Physiol..

[26] Koizumi M, Yada T (2008). Sub-chronic stimulation of glucocorticoid receptor impairs and mineralocorticoid receptor protects cytosolic Ca2+responses to glucose in pancreatic beta-cells. J. Endocrinol..

[27] Kadeba PI (2013). Regulation of store-operated calcium entry by FK506-binding immunophilins. Cell Calcium.

[28] Urbach V, Walsh DE, Mainprice B, Bousquet J, Harvey BJ (2002). Rapid non-genomic inhibition of ATP-induced Cl- secretion by dexamethasone in human bronchial epithelium. J. Physiol. (Lond.).

[29] Verrière VA (2005). Rapid effects of dexamethasone on intracellular pH and Na+/H+exchanger activity in human bronchial epithelial cells. J. Biol. Chem..

[30] Prota LFM (2012). Dexamethasone regulates CFTR expression in Calu-3 cells with the involvement of chaperones HSP70 and HSP90. PLoS One.

[31] Ghaisas M (2010). Preventive effect of Sphaeranthus indicus during progression of glucocorticoid-induced insulin resistance in mice. Pharm. Biol..

[32] Sullivan PW, Ghushchyan VH, Globe G, Schatz M (2018). Oral corticosteroid exposure and adverse effects in asthmatic patients. J. Allergy Clin. Immunol..

[33] Tamez-pérez HE, Quintanilla-flores DL, Rodríguez-gutiérrez R, González-González JG, Tamez-Peña AL (2015). Steroid hyperglycemia: prevalence, early detection and therapeutic recommendations: a narrative review. World J. Diabetes.

[34] Munemasa T (2018). Salivary gland hypofunction in KK-Ay type 2 diabetic mice. J. Diabetes.

[35] Sreebny LM, Yu A, Green A, Valdini A (1992). Xerostomia in diabetes mellitus. Diabetes Care.

[36] Itagaki K (2010). Dexamethasone stimulates store-operated calcium entry and protein degradation in cultured L6 myotubes through a phospholipase A(2)-dependent mechanism. Am. J. Physiol. Cell. Physiol..

[37] Storer CL, Dickey CA, Galigniana MD, Rein T, Cox MB (2011). FKBP51 and FKBP52 in signaling and disease. Trends Endocrinol. Metab..

[38] Fagerberg L (2014). Analysis of the human tissue-specific expression by genome-wide integration of transcriptomics and antibody-based proteomics. Mol. Cell. Proteom..

[39] Romanenko VG, Nakamoto T, Srivastava A, Begenisich T, Melvin JE (2007). Regulation of membrane potential and fluid secretion by Ca2+-activated K+channels in mouse submandibular glands. J. Physiol..

[40] Peña-Münzenmayer G (2015). Ae4 (Slc4a9) anion exchanger drives Cl- uptake-dependent fluid secretion by mouse submandibular gland acinar cells. J. Biol. Chem..

